# Single‐Cell and Spatial Multiomics: Applications for Diseases

**DOI:** 10.1002/mco2.70553

**Published:** 2025-12-14

**Authors:** Wentao Li, Chao Chen, Xin Zhu, Chenping Zhang

**Affiliations:** ^1^ Key Laboratory of Head & Neck Cancer Translation Research of Zhejiang Province Zhejiang Cancer Hospital Hangzhou China; ^2^ Innovation Center of Chinese Medicinal Crops Horticultural Research Institute Zhejiang Academy of Agricultural Sciences Hangzhou China; ^3^ Zhejiang Cancer Research Institute Zhejiang Cancer Hospital Hangzhou China

**Keywords:** deep learning, multiomics, precision medicine, single‐cell omics, spatial omics

## Abstract

Complex and dynamic networks of molecules are involved in human diseases. ​Single‐cell and spatial multiomics approaches have created new avenues for understanding the pathogenesis and diagnosis of diseases. Cell connections and characteristics in diseases may be examined more thoroughly by integration single‐cell and spatial multiomics. In this paper, we first reviewed the single‐cell and spatial multiomics approaches. Subsequently, the use of single‐cell and spatial multiomics to comprehend the mechanisms of human diseases, such as cancer (head and neck squamous cell carcinoma), neurodegenerative diseases, and aging, was discussed. Furthermore, we outline how deep learning approaches are now being applied to single‐cell and spatial multiomics data analysis in an effort to better define the pathogenic alterations upstream and the downstream molecular effects of diseases. Particularly, single‐cell and spatial multiomics are being utilized to help guide treatment plans, evaluate risks, and determine how they can affect precision medicine. Despite the relative youth of the field, the development of single‐cell coupled with spatial multiomics promises to provide a powerful tool for elucidating the pathogenesis of diseases.

## Graphical abstract

Schematic diagram of different single‐cell and spatial multiomics methods to describe the tissue layout of human diseases.

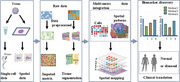

## 1 Introduction

Biological processes, such as the emergence of human diseases, are influenced by a wide range of environmental stimuli and include highly dynamic and interacting systems of molecular layers, including proteins, metabolites, mRNA transcripts, and genetics. Omics approaches have been extensively employed in the last several decades to identify processes and biomarkers for a variety of diseases [1, 2]. Different omics techniques, such as proteomics and metabolomics, are used to monitor data on proteins and metabolites at different biological stages [3]. For instance, metabolomics holds a unique role in multiomics research and is receiving more attention in the integrative analysis [4]. Metabolites are the downstream output of biological processes that carry genetic and environmental marks. They are also known as the link between genotype and phenotype [5] and they have been linked to a variety of disorders, such as ischemic stroke [6], type 2 diabetes [7], and cancer [8]. Moreover, they also carry integrated biological and medical signals in easily accessible biofluids (e.g., blood, urine), making them promising biomarker candidates. Integrating metabolomics with other omics, such as genetics, has proven to reveal the connections of metabolic individuality and disease pathway [9, 10]. Omics approaches have provided comprehensive knowledge about cellular components and biomolecules including genes, RNA, proteins, and metabolites. Genomics, transcriptomics, proteomics, and metabolomics are increasingly producing promising results, and these multiomics approaches are complementary, especially if different omics approaches are applied to the same patient. Therefore, we are now obtaining a comprehensive molecular understanding of disease across multiple types of omics data, leading to the development of precise medicine treatments.

**FIGURE 1:**
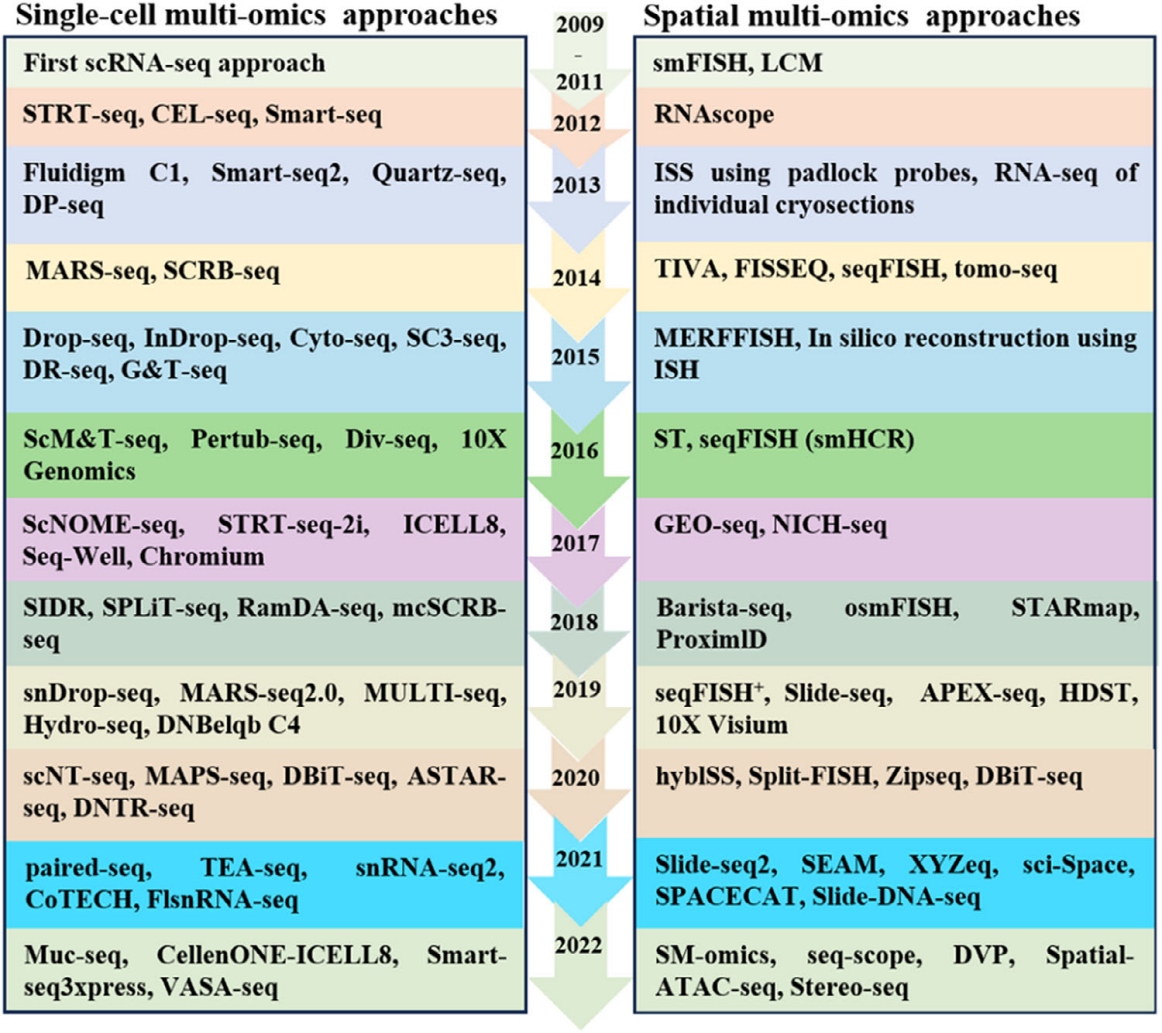
Timeline of single‐cell and spatial multiomics approach (by FigDraw). The development of single‐cell and spatially multiomics technologies between 2009 and 2022. Researchers can analyze molecular expression profiles at single‐cell resolution and also determine their spatial coordinates within tissues. *Abbreviations*: STRT‐seq: single‐cell‐tagged reverse transcription sequencing; CEL‐seq: cell expression by linear amplification and sequencing; DP‐seq: differential privacy sequence; MARS‐seq: massively parallel single‐cell RNA sequencing; SCRB‐seq: single‐cell RNA barcoding sequencing; SC3‐seq: single‐cell consensus clustering sequencing; DR‐seq: direct RNA sequencing; G&T‐seq: genome and transcriptome sequencing; ScM&T‐seq: simultaneous single‐cell methylome and transcriptome sequencing; Div‐seq: single‐nucleus RNA‐sequencing; ScNOME‐seq: single‐cell multiomics sequencing; SIDR: simultaneous isolation of genomic DNA and total RNA; SPLiT‐seq: split‐pool barcoding sequencing; mcSCRB‐seq: single cell RNA barcoding sequencing; snDrop‐seq: single nucleus RNA sequencing; MAPS‐seq: metagenomic plot sampling by sequencing; DBiT‐seq: deterministic barcoding in tissue for spatial omics sequencing; ASTAR‐seq: automated single‐cell transcriptome and chromatin accessibility sequencing; DNTR‐seq: direct nuclear tagging and RNA sequencing; CoTECH: combinatorial barcoding and targeted chromatin release; FlsnRNA‐seq: protoplasting‐free full‐length single‐nucleus RNA profiling in plants; VASA‐seq: vast transcriptome analysis of single cells by dA‐tailing.

The building blocks of all biological, morphological, structural, and functional activity as well as the foundation of all living activities are cells. Life forms and their associated tissues exhibit highly structured configurations made up of various cell types [11]. Traditional research techniques, including histological staining, differentiate these cells types based on their shape and position, the presence of particular molecular markers that indicate cellular identity, and the functions attributed to them within the tissue, whether established or presumed. Nonetheless, the relatively small number of known molecular markers for certain cell types and the inability to infer cellular activities from gene expression alone are major factors contributing to the limits of these approaches. The aspiration to systematically investigate intricate biological processes with greater resolution has resulted in the development of various methods, such as multidimensional analyses at the level of individual cells [12]. The emergence of single‐cell RNA sequencing (scRNA‐seq) began with the work of Tang et al. in 2009, which for the first time achieved transcriptome analysis of a single cell [13]. The creation of Smart‐seq in 2013, which increased transcript coverage and sensitivity, and Drop‐seq in 2015, which enabled high‐throughput profiling of thousands of cells at once, are examples of later technical achievements. The commercialization of the 10× Genomics Chromium system in 2016 further democratized scRNA‐seq, making it accessible to a broader research community. The launch of the Human Cell Atlas project in 2017 marked the beginning of a new phase in single‐cell biology, aiming to map all human cells. In 2019, *Nature Methods* selected single‐cell multiomics as the Technology of the Year, while in 2020, spatial transcriptome technology received the same accolade [14] (Figure 1). Current advancements in single‐cell multiomics and spatial multiomics technologies enable researchers to not only analyze molecular expression profiles at cellular resolution but also determine their spatial coordinates within tissues, while simultaneously mapping gene expression patterns across specific anatomical locations [15]. Emerging applications of these new technologies in deciphering the dynamic pathophysiological processes underlying metabolic disorders have significantly enhanced our systems‐level comprehension of disease mechanisms, while establishing critical molecular foundations for developing precision medicine frameworks.

In cancer research, developmental biology, and neurology, single‐cell and spatial multiomics approaches have been innovatively used, especially for evaluating tumor heterogeneity and T cell infiltration [16]. Single‐cell multiomics technology may use single‐cell genomic, transcriptome, protein, and metabolome data to provide more detailed insights into the properties and regulatory processes of cells. Utilizing spatial multiomics technology, we may get a better understanding of the molecular origins of diseases and the spatial variation of disease molecules [17]. Single‐cell and spatial multiomics technologies can aid in our comprehension of the molecular causes of diseases. Nevertheless, there are still several restrictions on the use of single‐cell and spatial multiomics technologies in treating diseases. To guarantee the correctness and dependability of these methods, more fundamental study is required. Standardization and standardized data interchange procedures, technological dependability, and other problems like data analysis and algorithm optimization are only a few of the many obstacles and problems that must be overcome. These issues will eventually be resolved, and the pertinent study findings will be further enhanced, with the ongoing development and enhancement of linked technologies. Our knowledge of diseases will also be improved by the use of single‐cell and spatial multiomics.

In this review, we first reviewed the single‐cell and spatial multiomics approaches. Subsequently, we reviewed the application of single‐cell and spatial omics in human diseases, such as cancer (head and neck squamous cell carcinoma [HNSCC]), neurodegenerative diseases, and aging studies, and analyzed the main research information of single‐cell and spatial omics. In addition, we reviewed recent applications of deep learning methods to single‐cell and spatial omics data analysis, which aids in the characterization of pathogenic alterations and molecular influences in diseases. Finally, we discussed the role of single‐cell and spatial omics research in personalized medicine. Furthermore, the application of single‐cell and spatial multiomics is influencing precision medicine by directing treatment strategies and risk evaluation. It is evident that omics is heading toward single‐cell and spatial analysis, which is unquestionably one of the most potent instruments available to us at the present. This will enable us to analyze diseases in greater depth and with greater precision.

## 2 Single‐Cell and Spatial Multiomics Approaches

This section will analyze the principle, progress, and limitations of single cell and spatial multiomics approach from three dimensions: transcriptome, proteome, and metabolomics, focusing on the application of 10× Genomics Visium platform and MALDI–mass spectrometry (MS) imaging in cancer, such as HNSCC, neurodegenerative diseases, and aging research.

### 2.1 Transcriptomics: Single‐Cell Transcriptomics and Spatial Transcriptomics

Genetic mutations and changes that might occur during cell division and proliferation are the cause of genetic heterogeneity [18, 19, 20, 21]. Whole‐genome sequencing may be used to identify recombination, aneuploidies, structural variations, and mutations in the genome. The integration of transcriptional data with results from other investigations, such as those that examine genomic stability and chromatin accessibility, has facilitated a deeper comprehension of mechanistic research. Analysis of genomes and alterations in their activities at the cellular level is now feasible because to single‐cell genomic sequencing [22, 23, 24, 25]. Because cellular genomic heterogeneity that may be implicated in disease processes can now be consistently revealed by somatic mutations, such as copy number variations (CNVs) and single nucleotide variations (SNVs) [26, 27].

DNA mutations can occur in oocytes and sperm, or somatic and germ cells. A significant and unexpected level of genomic variety that contributes to the gamete's genome diversity has been shown by recent research that have generated recombination maps and mutation rates for individual human sperm and oocytes. Apart from germline mutations, aging and degenerative disorders are frequently linked to somatic tissue mutations. Although this result is debatable and the potential biological ramifications are yet unknown, megabase CNVs seem to be found in a significant portion of single human neurons from healthy individuals. The study of single‐cell somatic mutation documentation is a rapidly deep learning expanding subject. Still, little is understood about the underlying mechanisms and how they could work in both healthy and diseased conditions.

Cells of interest are sequenced to obtain transcriptome snapshots from which developmental states are inferred by comparing them to reference cells to perform fate mapping. Nevertheless, these data lack long‐term lineage information spanning several cell generations and are often only meaningful for the sampling time. To ascertain ancestry across several generations, single‐cell DNA analysis is required. Cell lineage trees may be produced by using genome sequencing techniques to find random somatic mutations that have accumulated in a single cell. Due to the rarity of somatic mutations, this approach naturally has low sensitivity and resolution. Using CRISPR–Cas9 genome editing to create genomic scars in conjunction to scRNA‐seq (Single‐cell RNA Sequencing) might significantly lessen this restriction [28, 29]. To do this, a technique for identifying the fast accumulation of mutations in target cells while also tracing their genealogical paths has been developed.

These investigations employ fresh frozen or FFPE tissues, depending on the procedure. The two primary techniques for spatial transcriptomics are fluorescent in situ hybridization and next‐generation sequencing (NGS) [30, 31, 32]. Prior to sequencing, transcripts are encoded with positional information using the NGS approach. mRNA is extracted from the tissue overlay on chips using spatially barcoded oligos on the 10× Genomics Visium device to provide objective spatial transcriptomic data [33]. The mRNA is then analyzed in preparation for sequencing. However, the effective spatial resolution is not at the single‐cell level because of the limited number of recorded transcripts, which means that transcripts from several nearby cells are combined for further analysis. To extract RNA from tissue samples, other spatial transcriptome methods use spatial barcode probes attached to beads [34]. About 50% of scRNA‐seq is captured by slide‐seq V2, which has a higher RNA capture efficiency than slide‐seq [35]. On the high‐definition spatial transcriptomics (HDST) technique, transcripts are recorded using fluorescent spatial indexing beads located on microwells, and the locations of transcripts in tissue samples are identified by fluorescence signals. Sequencing data are mapped to fluorescence data once the barcoded RNA has been sequenced. Individual transcripts may be sequenced cyclically to increase resolution. For both slide‐seq and HDST, the size of the beads and the effectiveness of RNA transcript capture have an impact on the resolution of spatial information. The diameter of the HDST beads is around 2 µm, whereas the slide‐seq beads have a diameter of 10 µm. HDST provides higher resolution and captures more targets than slide‐seq.

The development of spatial transcriptomic approaches has enhanced transcriptome capture efficiency, enabling the acquisition of images with sub‐µm resolution. Tissue is affixed to an RNA‐capturing array in Seq‐Scope, which has a dense configuration of barcoded clusters [36]. To make cDNA for NGS analysis, templated mRNAs are extracted from the tissue. Even though low‐abundance copy transcripts are not yet identifiable, this approach has been used to analyze the transcriptomes of solid tumors at almost single‐cell resolution and mouse embryonic development.

Combining interactive in situ hybridization or in situ sequencing with high‐resolution imaging allows image‐based techniques to get subcellular spatial resolution and perhaps generate transcriptome data throughout the whole genome. Large probe sets, complex image processing procedures, and iterative workflows are typically required for these technically challenging approaches. Probes hybridize preselected RNA targets to facilitate reverse transcription during in situ sequencing procedures. Rolling circle amplification, sequencing by synthesis, sequencing by ligation, or sequencing by hybridization are all possible read‐out techniques [37]. Through reverse transcription and roll‐up amplification, fluorescence in situ sequencing, an untargeted gene profiling method, creates cDNA amplicon nanorods with a diameter of 200–400 nm inside the cell. To obtain in situ transcriptome data, the amplicons were sequenced using a technique called assisted oligonucleotide ligation and detection [38]. Spatially accurate transcript amplicon readout mapping, which blends hydrogel tissue chemistry techniques with tailored signal amplification, has enabled the acquisition of personalized in situ transcriptomics pictures.

A wide range of commercial and academic systems, each with unique advantages and disadvantages in terms of spatial resolution, molecular capture efficiency, sensitivity, tissue compatibility, and throughput, have emerged as a result of the quick development of spatial transcriptomics. Whether whole‐transcriptome discovery or targeted, high‐sensitivity quantification is needed, as well as whether the analysis must be at a multicellular, cellular, or subcellular resolution, are all important factors to consider when selecting a platform. To provide a clear overview, we objectively compare three prominent platforms in Table 1.

**TABLE 1:** Comparison of mainstream spatial transcriptomics platforms.

Characteristics	10× Genomics Visium	NanoString GeoMx DSP	STOmics (Stereo‐seq)
Principle of space capture	Random capture based on arrays	Region selection based on fluorescence	High‐density array based on DNA nanospheres
Resolution	55 µm (capture area diameter), ∼5–10 cells	Cellular level to subcellular level	Submicron scale (500 nm), single‐cell/subcellular level
Flux	The entire tissue section (∼6.5 × 6.5 mm)	Multiple user‐defined ROIs on tissue sections	An extremely large tissue area (∼13 × 13 cm)
Organizational compatibility	Fresh frozen and FFPE	Fresh frozen and FFPE	Mainly fresh frozen
Advantage	Standardization of operating procedures Intuitive organizational view	Morphological guided flexible ROI selection High sensitivity	Extremely high spatial resolution Single‐cell level analysis
Challenge	Resolution limited to the multicellular level The number of gene detections is limited by the capture point.	ROI selection introduces human bias. Need prior knowledge or pre‐experimentation	The amount of data is extremely large. The data analysis process is more complex.

### 2.2 Proteomics: Single‐Cell Proteomics and Spatial Proteomics

In single‐cell proteomics approaches, the primary tool for sorting and classifying cellular phenotypes is fluorescence‐activated cell sorting, also known as fluorescence flow cytometry. A single‐cell analysis of multiple functional proteins is possible through the measurement of surface marker proteins. Two light scatter metrics that help with size‐based discrimination between various cell types are forward and side scatter, which may be used to phenotypically classify cell types, especially immune cells. Additional factors connected to other membrane proteins that have fluorescent antibodies attached to them can also be used for this. Although single‐cell proteomics techniques are still in their infancy, a recently discovered protein mass spectrometry (MS) approach has the potential to increase single‐cell protein measurements to 34 [39]. Mass cytometry, which employs antibodies attached to specific isotopes, is paired with target epitope binding on and within cells. It is possible to monitor 34 parameters per cell at the same time by spraying single‐cell droplets over inductively linked argon plasma. A thorough proteome study of a single‐cell precise cell phenotype has been made possible by this technology, which provides a methodical illustration of the signaling behaviors that underlie the signaling responses specific to different cell types in complex organs. Another high‐throughput method for single‐cell proteomics is MS, which concurrently tackles the problems of locating and quantifying peptides in a single cell and transferring the proteome to the MS with the least amount of protein loss [40]. Alternatively, antibodies attached to DNA barcodes are used to identify cellular proteins, and these are evaluated in combination with a single‐cell transcriptome using a modified scRNA‐seq approach [41]. By combining these advanced and highly multiplexable techniques with scRNA‐seq approaches, accurate protein quantification may be accomplished.

In response to the notion that several proteins may be recognized in a single staining step that arose from traditional IHC, highly multiplexed spatial proteomic detection was created. Regarding cell throughput, spatial resolution, temporal dynamics, and the number of chemical targets, all of the methods are comparable. In fluorescence‐based approaches, fluorescently tagged primary antibodies are periodically added to and removed from the target FFPE tissue in a procedure called iterative picture capture. The design of oligonucleotides or the usage of fluorophores determines how well spatial detection using these iterative techniques works. Throughout the repeated detection process, the antibodies are attached to fluorophores with low spectrum overlap, indexing oligonucleotides, amplified oligonucleotides, or orthogonal DNA concatemers to give specificity and sensitivity. The MACSima imaging system and tissue‐based circulating immunofluorescence employ mild methods like photobleaching with specific antibodies or a specialized removal reagent to bleach or inactivate the label. Using combinatorial analysis, the multiomics single‐scan experiment connects secondary probes to the combinatorial fluorophore labels for imaging [42]. Microscopy and fluorescent lifetime imaging are used to record spatial outcomes. Three‐dimensional tissue images are produced by mapping the spectral and temporal information to the original photos using phasor analysis after the fluorescence spectrum and lifetime data are decoded using a machine learning‐based decoding approach.

Spatial proteomics methods based on metal tagging include imaging MS, cytology, and multiple ion beam imaging [43, 44]. These techniques include dyeing tissue samples with metal‐conjugated antibodies and identifying target proteins using MS, which measures the quantity of isotopic reporter masses released from the tissue after laser or ion beam ablation. Imaging mass cytometry (IMC) uses a single laser to ablate tissue. Multiplexed ion beam imaging (MIBI) two source ion beams provide a higher spatial resolution than IMC [45, 46]. Using tumor samples from preclinical and clinical research, IMC and MIBI have both made tremendous progress in our knowledge of the intricate cellular structure of TIME and its involvement in carcinogenesis [47, 48]. Both mRNA and protein may be found in the same tissue using multiplexed fluorescence and metal‐based tagging techniques. The goal of codetection techniques is to simultaneously show a cell's phenotypic and genotypic characteristics [49].

These methods entail labeling tissue samples with metal‐conjugated antibodies before ablating the tissue with a laser (IMC) or primary ion beam (MIBI). MS is used to quantify the released isotopic reporters, allowing for highly multiplexed protein detection. MS is used to quantify the released isotopic reporters, allowing for highly multiplexed protein detection. In particular, MIBI‐TOF provides great sensitivity and spatial resolution. CODEX (codetection by indexing), an alternative method to highly multiplexed spatial proteomics, uses a library of DNA‐barcoded antibodies and successive cycles of hybridization, imaging, and dye inactivation to visualize dozens of proteins at once on a standard fluorescence microscope [50]. This method is particularly powerful for deep phenotyping of the tumor microenvironment (TME) and immune contexture.

### 2.3 Metabolomics: Single‐Cell Metabolomics and Spatial Metabolomics

Finding the cell types with distinct metabolite patterns is the goal of single‐cell metabolomics, which attempts to explain tissue heterogeneity [51]. The difficulty of identifying short‐lived proteins that are not chemically stable in vitro using MS techniques makes this procedure particularly challenging. Techniques for single‐cell metabolomics are still in their infancy, and the ones that are available now have extremely low sensitivity and a lot of technical noise. Nonetheless, the development and enhancement of existing MS techniques have enabled the detection of the trace amounts of metabolites present in individual cells. The ongoing controversy regarding the ability of small molecules to alter epigenetic profiles and, by extension, transcriptomic profiles at the single‐cell level will be resolved if these molecules, metabolites, and associated signaling events are effectively incorporated into experiments or paired with data from epigenetic measurements [52, 53].

Multiomics integration at the single‐cell level is beginning to be made possible by recent technological developments that measure metabolites concurrently or sequentially with other molecular layers. For example, metal‐tagged antibodies or chemical probes that target important metabolic enzymes or metabolites are used in CyTOF‐based single‐cell metabolic profiling, which enables the simultaneous measurement of proteins and metabolic processes inside millions of single cells using mass cytometry [54]. Another cutting‐edge approach, live‐seq, enables direct linkage of transcriptional states to the cellular metabolic footprint by combining live cell transcriptome profiling from cytoplasmic biopsies with posthoc MALDI–MS analysis of the same single cell [55]. Moreover, hundreds of metabolites from individual cells may now be detected using very sensitive single‐cell MS instruments. These metabolomic profiles, when matched with data from scRNA‐seq or scATAC‐seq performed on sibling cells from the same population, provide significant insights into the regulatory relationships between metabolism, gene expression, and epigenetics.

A reliable method for the multiplexed analysis of proteins, natural products, and metabolic byproducts is MS [56]. Since conventional MS methods cannot provide spatial information, other ionization approaches have been employed in MS‐based imaging schemes [57]. Up till now, several techniques for separating and gathering molecules from individual cells in both natural and vacuum environments have been documented. Among these are the ion beam [58], laser [59], and probe [60] sampling techniques. The single‐cell isolation approach is the most crucial stage since a poorly executed one might cause environmental disruption and provide erroneous metabolic profiles [61, 62]. Ion beam and laser techniques are now the most used methods for ionization and sampling under vacuum circumstances. The ionization and sampling procedures for these techniques take place simultaneously. Ion‐beam based methods such time‐of‐flight MS and nanoscale secondary ionization MS can provide high resolution (100 nm to 1 µm) [63]. These techniques provide outstanding sensitivity and spatial resolution, making them valuable for intracellular research and single cell imaging [64, 65].

Secondary ionization MS‐based methods are often more energetic, but they also frequently produce a large number of fragments, which makes processing data for the majority of biomolecules more challenging. MS using MALDI is the main laser‐based method for examining individual cells. The way the laser beam interacts with organic matrix molecules to initiate the analyte's ionization and desorption forms its basis. This technique has remarkable salt tolerance and offers high measurement sensitivity and throughput [66, 67]. Additionally, MALDI–MS can produce high‐fidelity results that precisely represent the natural distribution of cellular analytes when combined with a flash‐freezing sample preparation procedure [68]. Due to the vacuum working conditions, secondary ionization MS and MALDI‐based procedures, with their advanced equipment and wide range of applications, need careful sample preparation. In contrast to cells in their native biological environment, these conditions may change the chemical composition of the cells. For metabolites that have a high turnover rate, this unique characteristic is even more beneficial [69]. A number of creative approaches have been developed as a result of workplace flexibility. Nanoelectrospray ionization is the primary source of ionization in these procedures, which often include distinct phases for sampling and ionization.

## 3 Unravelling Human Diseases With Single‐Cell and Spatial Multiomics Approaches

**FIGURE 2:**
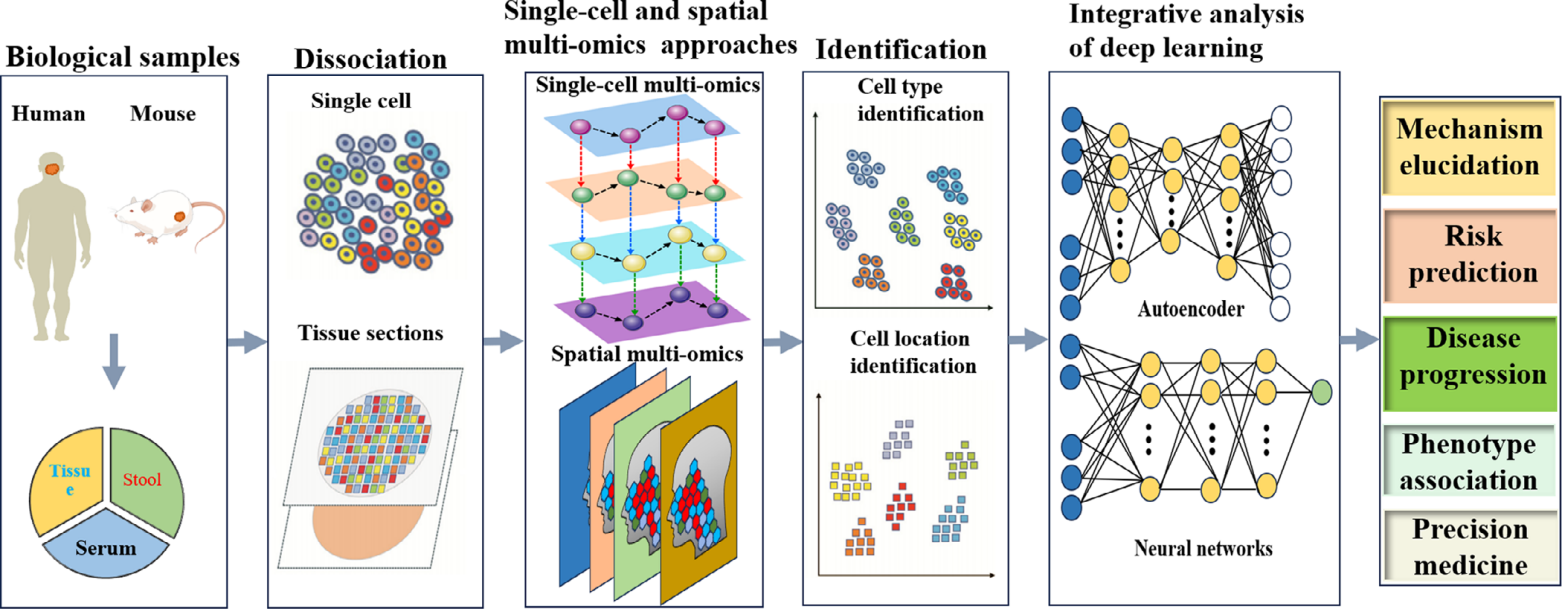
Brief schematic diagram of the single‐cell and spatial omics approaches process (by FigDraw). These techniques have played a significant role in enhancing our understanding of the composition of complex cell types and their corresponding cellular functions in living organisms.

Analyzing cellular processes in depth may be crucial to understanding the pathophysiology of human diseases. It may be possible to identify important pathways by combining different omics approaches. A state‐of‐the‐art method called multiomics makes it possible to analyze several molecular compartments and their alterations at the same time and with great detail. The resultant information has revolutionized biology and medicine by enabling integrated analysis for a better knowledge of how molecules interact and, consequently, enhancing diseases prognosis. At the single‐cell level, omics approaches are becoming more and more practical. A shift in viewpoint made possible by this high resolution makes it possible to identify cell‐specific pathways and interactions. Molecular signals can now be found using these methods to investigate the pathophysiological mechanisms behind diseases (Figure 2).

### 3.1 The Application of Single‐Cell and Spatial Multiomics in Cancer: HNSCC as an Example

Due to the heterogeneity of HNSCC, conventional sequencing methods are limited to analyzing whole tumors and are unable to reveal information on the heterogeneity of individual cells [70]. Tumor heterogeneity arises from variations in the genetic and molecular properties of individual cancer cells within the same tumor tissue, which are caused by varying degrees of cell differentiation [71]. Tumor heterogeneity interacts with the TME to encourage cell subtype differentiation and metastasis, hence contributing to the development of cancer [72, 73]. The process of sequencing a single cell at the transcriptome or genome level to provide genomic, transcriptomic, or other information about that specific cell can be used to identify changes in cell populations and their evolutionary relationships [74]. Further examination of the complex heterogeneity of HNSCC and improvements in diagnosis, prognosis, and treatment monitoring are made possible by spatial omics research's improved ability to pinpoint cell‐cell interactions and identify heterogeneous cells inside individual malignancies [75]. This provides an opportunity for understanding inter‐ and intratumor heterogeneity. Here, the study on single‐cell and spatial multiomics in HNSCC was reviewed, and we provided a reference for researchers to better understand the tumor's characteristics from a variety of angles, look into its potential for clinical application, and direct focused treatment.

#### 3.1.1 Single‐Cell and Spatial Multiomics Explore the Cells Type and Function of HNSCC

The most prevalent cell type in tumor tissue and one of the main cellular elements of the immune response, T cells have the ability to eradicate tumor cells [76, 77, 78]. Since Allison et al. found in 1996 that cytotoxic T lymphocyte‐associated antigen 4 (CTLA4) suppresses T cells immunological systems, immunotherapy medications—most notably immune checkpoint inhibitors (ICIs)—have become widely used and are now life‐saving treatments for patients with tumors [79]. Many ICIs, such as CTLA4 and PD1/PDEEP LEARNING1, have been used in cancer immunotherapy [80, 81]. Nevertheless, the effectiveness of immunotherapy in treating HNSCC patients is still lacking. As a result, more studies are being carried out to thoroughly analyze TME using the single‐cell approach.

One of the most effective treatment approaches in cancer today is immunotherapy, which works by increasing immune cell production or by reactivating endogenous immune responses that the tumor has suppressed through the use of ICIs [82]. Due to the crucial role of antitumor immunity and immunotherapy, many analyses have focused on T cells. ​The T cell population in the tumor tissue had a much larger proportion of CD8^+^ cells relative to the surrounding normal tissue [83]. According to single‐cell sequencing, enlarged clonotypes—characterized by clonal sizes of two or more cells and up to 162 cells—are more common in the interior of the tumor than in the normal mucosa nearby and are concentrated in exhausted T cells [84]. ​Immunohistochemistry confirmed that programmed death 1 (PD‐1) expression is enhanced in OSCC infiltrating cells compared with normal lymphoid tissue cells. Regulatory T cells (Tregs) are significantly more prevalent in tumors than in nearby normal tissues within the CD4^+^ cell population, which supports the immunosuppressive character of TME [85, 86]. Features linked to CD8^+^ and CD4^+^ cell reduction in the HNSCC dataset are linked to shorter OSCC [86]. Of the 12 patients, it was projected that eight of them would have macrophages as their primary source of PD‐L1 for interaction with PD‐1 on CD8^+^ cells, and that just two would have epithelial cells. High levels of PD‐L1 expression on macrophages and their apposition to T cells were verified by flow cytometry and multispectral fluorescence microscopy. This confirmed clinical findings that a composite PD‐L1 score that took into account both tumor cells and macrophages was the strongest indicator of ICI response [87].

In a study conducted by Moreno‐Nieves et al., innate lymphoid cells (ILCs) were identified as CD56^+^ and/or CD127^+^ cells from the main tumors, lung metastases, and peripheral blood of eight patients with hepatocellular carcinoma (HNSCC). The purpose of the study was to investigate the contentious role of natural killer (NK) cells in tumor management [88]. The intraepithelial ILC cluster 1 (ieILC1) had the highest cytolytic activity among intratumoral NK‐related cell subsets, although gene expression patterns indicated that NK‐2 cells were diseased. These two cell types may have marked the ends of two distinct circulating NK cell development trajectories, according to pseudotime analysis. IL‐15 and TGF‐β induced the ieILC1 markers CD49a and CD103 to be upregulated in a subset of circulating NK cells in coculture with HNSCC cells, whereas cells that remained CD49a‐resembled NK‐2 cells. When exposed to tumor cells or other stimuli, in vitro developed ieILC1‐like cells generated more interferon γ and tumor necrosis factor α, degranulated more, and killed tumor cells more efficiently than NK‐2‐like cells. In vitro differentiated CD49a cells greatly outperform their CD49a counterparts in controlling tumor development when injected subcutaneously with HNSCC cells into immunocompromised mice. Therefore, it was proposed that ieILC1 cells had the strongest anticancer activity among innate lymphoid/NK cells in the TME [88].

Examination of the primary tumor, lymph node metastases, normal tissue, and precancerous oral leukoplakia of 23 patients revealed that some of the leukoplakia epithelial cells had CNVs. This implies that leukoplakia's CNV‐driven expression of recombinant human tumor protein 63 and recombinant sodium/potassium transporting ATPase subunit beta‐3 is essential to the development of HNSCC [89]. A study conducted on matched normal, dysplastic, and tumor cells obtained from OSCC biopsies revealed a progressive rise in the expression of genes related to pathways including mechanistic target of rapamycin complex 1‌ and EMT. Interestingly, vascular endothelial growth factor A was also found to be a cancer‐initiating agent in the study [90]. Furthermore, different fibroblast subtypes have been reported to promote malignant transformation [91]. According to trajectory studies, the fibroblasts in leukoplakia exhibit features more similar to those found in malignant tissue than in normal tissue, suggesting that they may have attained cancer‐associated fibroblasts (CAF) characteristics. In leukoplakia Tregs, collagen type I α1 enhances the expression of leukocyte‐associated immunoglobulin‐like receptor 2, a soluble collagen receptor that triggers proinflammatory processes that foster a milieu conducive to tumor growth. Factors facilitating the growth of HNSCC have been identified as fibroblast‐malignancy cell interactions via the receptors LGALS7BCXCL8 and COL1A1–CD44 [89]. Through the use of scRNA‐seq, scTCR‐seq, and trajectory analysis on seven pairs of original tumors and cervical lymph node metastases from patients with HNSCC, a unique subpopulation of premetastatic cells was discovered. There were several paths taken by CD8^+^ cell clones that led to SRY‐related high mobility group box 4‐mediated T cell dysfunction. One path showed a gradual loss of naïve markers and an increase in dysfunctional markers [92].

#### 3.1.2 Single‐Cell and Spatial Multiomics Were Used to Investigate Therapeutics and Potential Targets for HNSCC

All types of therapy can cause primary and secondary resistance, which is the primary cause of cancer‐related mortality [93]. ​For example, Osman et al. employed scRNA‐seq to investigate cisplatin resistance in human HNSCC cell lines and discovered that cisplatin resistant cells exhibit enhanced Forkhead box O signaling, TP53 signaling, aging, and the cell cycle [94]. Using scRNA‐seq and multiplex immunohistochemistry, it was possible to assess the amount of infiltrating immune cells before and after therapy in eight patients with HNSCC. The first‐line treatment regimen used in the study for patients with advanced nasopharyngeal cancer is “TPF (paclitaxel, cisplatin, 5‐fluorouracil) induction chemotherapy combined with cetuximab”. Treatment responses may be estimated based on nonmalignant cell subtypes categorized as “tumor suppressor” or “tumor promoter” by using the cell type deconvolution approach of the scRNA‐seq dataset and classifier training [95]. An analysis of the publicly accessible scRNA‐seq dataset was conducted to look into the radiation sensitivity heterogeneity in OSCC cells. The findings indicate that radiation sensitivity is linked to the atypical subtype, while radiation resistance is connected with the coexpression module, which is present in both the classical and basal subtypes [96]. Radiation‐resistant tumors showed an increase in immune checkpoint interactions, which offered a theoretical foundation for a combined treatment of irradiation and immune checkpoint blockage for HNSCC. But thus far, clinical trials have not shown that this strategy works [97, 98].

In a mouse model, anti‐PD‐1 or anti‐CTLA4 treatment improved T cell differentiation toward a more active state. Tumor‐associated macrophage infiltration in large numbers into ICI‐resistant malignancies is facilitated by the production of CSF1 and VEGF in tumor cells [99, 100]. By examining scRNA‐seq from four tumors in patients with advanced NSCC both before and after ICI therapy, Obradović et al. assessed whether TME subgroups connected to CAF influence the clinical response to nivolumab. Two of the five CAF clusters are predictive of the nivolumab response, but the third is linked to immunological suppression [101]. Using scRNA‐seq of TILs extracted from tumor biopsies of six patients with HNSCC, one research examined the dynamics of CD4^+^ and CD8^+^ cells both before and after neoadjuvant treatment with bintrafusp. This treatment mostly stimulated worn‐out CD8^+^ TILs, which was linked to altered glutamine metabolism. It was proposed that blocking TGF‐β signaling may encourage treated TILs to leave the body and return to the bloodstream, perhaps leading to better patient outcomes [102].

The substantial variability of HNSCC makes molecular‐level research challenging. It is necessary to consider both chemical data and the spatial distribution of molecules inside the TME to comprehend molecular modifications. Spatial measurements help us better understand the structure and function of incredibly complex biological systems [103]. The exploration of heterogeneity in HNSCC is essential to elucidating a more profound understanding of the relevant processes and improving the accuracy of cancer disease diagnosis and treatment. Spatial metabolomics offers early detection and fast treatment to halt the growth of cancer cells by analyzing certain chemical fingerprints in tissue samples. This technique has a wide range of therapeutic applications, such as identifying histopathological abnormalities, looking for metabolic markers, diagnosing diseases early, assessing the likelihood of metastasis, and accelerating the drug discovery process [104]. Moreover, the potential of spatial metabolomics for an early diagnosis within the distinct setting of HNC is yet in its early stages. By separating tumor from healthy tissue sections in patients with salivary gland malignancies using MALDI–MSI, Sommella et al. discovered chemical changes associated with the tumor's energy requirements [105]. Using MALDI–MS analysis to compare lipid and protein biomarkers, Katarzyna et al. addressed the tissue heterogeneity of squamous cell carcinoma and successfully distinguished oral cancer from normal mucous membranes, demonstrating the use of both domains as biomarkers [106]. By combining spectral principal component analysis and linear discriminant analysis with DESI–MSI, Cedric D'Hue et al. were able to effectively differentiate oral tongue squamous cell carcinoma from adjacent normal epithelium [107].

In conclusion, the recent development of single‐cell and spatial multiomics approaches has led to an increase in their application in HNSCC research. Thanks to these two technologies, researchers now have a more complete understanding of the processes behind the development and progression of HNSCC. First, single‐cell omics technology can aid researchers in comprehending how the expression profiles of various cell types alter when HNSCC develops. Second, research on HNSCC also heavily relies on spatial‐omics technologies. Applying these two technologies is still fraught with difficulties, though, including the multifaceted and intricate nature of HNSCC, complicated preparation and data analysis, and possible biases and inaccuracies in current analytic techniques. Single‐cell and spatial multiomics technologies are often used in HNSCC research in a substantial way, but more promotion and optimization are required for better assistance in treating and preventing HNSCC. Tables 2 and 3 summarize the application of single‐cell and spatial multiomics approaches in preclinical and clinical experiments of HNSCC.

**TABLE 2:** Summary of the application of ​single‐cell and spatial multiomics approaches in preclinical experiments of HNSCC.

Species	Sequencing method	Sample	Platform	Main findings	References
Mouse	scRNA‐seq	Tissues	10× Genomics	TDO2^+^ myofibroblasts were more likely to possess the ability for chemotaxis toward T cells.	[91]
		Tissues	10× Genomics	Analysis of the interaction between premetastatic tumor cells and CD8 T lymphocytes reveal a role in immune regulation.	[92]
		Tissues	Illumina HiSeq	Restoring the wild‐type KEAP1 gene or lowering NRF2 made resistant cells more sensitive to CDDP and reduced distant metastases.	[94]
		Tissues	Illumina HiSeq 4000	CD8^+^ TIL differentiation is a strategy to improve ICB response in HNSCC.	[99]
		Tissues	Illumina NovaseqS4 PE150	Genetic alterations have limitations in stratifying cancers and suggest that assessing intrinsic cues of HNSCC, as well as the immune profile of TME, may help better predict ICI responses.	[100]
	Spatial transcriptomics	Tissues	10X Visium	The results provide novel evidence for a feedback loop between tumor‐associated macrophages and cancer cells in glucose‐deficient regions.	[108]

*Abbreviations*: TDO2^+^: tryptophan 2,3‐dioxygenase; CD8: cytotoxic T lymphocytes; KEAP1: Kelch‐like ECH‐associated protein 1; CDPP: cisplatin; ICB: immune checkpoint blockade; TME: tumor microenvironment; ICI: immune checkpoint inhibitors.

**TABLE 3:** Summary of the application of ​single‐cell and spatial multiomics approaches in clinical experiments of HNSCC.

Species	Sequencing method	Sample	Platform	Main findings	Ref.
Human	scRNA‐seq	Tissues and peripheral blood	10× Genomics	The transcription factor PRDM1 was found to transactivate TOX expression via a binding motif in the TOX promoter.	[86]
		Tissues and peripheral blood	Illumina NextSeq 500	Tumor‐associated macrophages are predicted to be major contributors to TME PD‐L1 and other immune checkpoint ligands.	[87]
		Tissues and peripheral blood	HiSeq 2500 high‐output v4 sequencer	The ieILC1‐like cell state in TME was identified as the NK cell phenotype with the highest antitumor activity.	[88]
		Tissues	Illumina HiSeq 4000	The identification of malignant cells in the fibroblast subpopulation of Galectin 7B and CXCL8 suggests that abundance in tumor tissue is associated with poor prognosis.	[89]
		Tissues	10× Genomics	Identified a subset of myofibroblasts that exclusively expressed tryptophan 2,3‐dioxygenase (TDO2)	[91]
		Tissues	10× Genomics	The role of SOX4 in mediating T cell failure was revealed.	[92]
		Tissues	Illumina HiSeq 2000	A binary classifier model was developed to identify gene modules and the classifier model as a reliable predictor of hypopharyngeal carcinoma treatment response.	[95]
		Tissues	10× Genomics	The functional importance of distinct HNCAF subsets in modulating the immunoregulatory milieu of human HNSCC	[101]
		Tissues	10× Genomics	The frequency of activated blood CD8 T cells, notably pretreated PD‐1‐positive KLRG1‐negative T cells, was strongly associated with an intratumoral pathological response.	[102]
		Tissues	SolariX XR 7T‐ESI/MALDI–Fourier‐transform ion cyclotron resonance mass spectrometer	While sphingomyelins and triacylglycerols, which are important components of the signaling pathway and energy generation, were logically decreased in tumor tissues, glycerophospholipids grew dramatically.	[105]
	Spatial transcriptomics	Tissues	MALDI–TOF/TOF ultrafleXtreme mass spectrometer	Tumor proteome and lipid group are important sources of biomarkers for oral malignant tumors.	[106]
		Tissues	DESI‐MS imaging	DESI‐MS accurately differentiated oral SCC from adjacent normal epithelium.	[107]
		Tissues	Illumina NextSeq500	There are protumor connections between macrophages and epithelial cells through the migration inhibitory factor (MIF)–CD74 axis.	[109]
	scRNA‐seq and spatial transcriptomics	Tissues	10× Genomics	Alterations in epithelial gene expression profiles are associated with different subpopulations of fibroblasts, monocytes, and regulatory T cells involved in reshaping the microenvironment.	[90]

*Abbreviations*: PRDM1: PR domain zinc finger protein 1; SOX4: CXCL8: chemokine (C‐X‐C motif) ligand 8; SRY (sex determining region Y)‐box 4; HNCAF: head and neck cancer‐associated fibroblasts; KLRG1: killer cell lectin‐like receptor g1.

### 3.2 The Application of Single‐Cell and Spatial Multiomics in Neurodegenerative Diseases

The loss of neurons and the myelin sheath is a common characteristic of neurodegenerative diseases. Numerous reports have linked the onset and development of various disorders to neuroinflammation and glial cell activation. These diseases have been linked to different cell subsets and a different distribution of glial cells, suggesting that the pathophysiology of neurodegeneration is linked to the functional alterations of particular cell subsets [110, 111]. Additionally, immunological heterogeneity is shown by single‐cell transcriptomics, which provides fresh indicators and possible treatment targets for these conditions.

#### 3.2.1 Revealing the Connection between Glia and the Pathogenesis of Neurodegenerative Diseases

As the most common neurodegenerative disease, the prevalence of Alzheimer's disease (AD) is increasing year by year [112]. Phosphorylated tau tangles and β‐amyloid (Aβ) buildup in the brain are common pathological features of AD. A buildup of Aβ may cause microglia to activate more quickly [113]. Through the use of computational clustering approaches, researchers have discovered several microglia subgroups in AD [114]. In the AD model, they saw a rise in neuropathology‐associated microglia and a reduction in homeostatic microglia (HM) [115]. The potential to prevent neurodegenerative damage was demonstrated by Keren et al.’s definition of a novel subpopulation of microglia known as disease‐associated microglia (DAMs) [116]. A vital part of the removal of Aβ and apoptotic neurons, DAMs often lived around the Aβ plaque.

Variations in APOE and TREM2, which are often regarded as the main genetic risk factors for AD, may have an impact on the microglial response to Aβ [117, 118]. DAMs have elevated APOE expression, indicating APOE's role in immune response and Aβ clearance [119]. The receptor triggering receptor expressed on myeloid cells 2 is encoded by TREM2, and when combined with APOE, it can help microglia identify and phagocytize Aβ [120]. Deficits or variations in TREM2 significantly raise the probability of developing AD and have important effects on Aβ clearance. Single‐cell investigation of DAMs in a study by KerenShaul et al. showed that these cells are produced from HM via a two‐step process, the second of which depends on TREM2. According to this, TREM2 may be a regulator that initiates DAM activation [116]. Moreover, Silvin and associates used single‐cell information from six distinct investigations in mouse brains to methodically define the role of DAMs. They discovered that the disease inflammatory macrophages (DIMs) and TREM2‐dependent DAMs were two different populations of DAMs in Keren's dataset [121]. In AD‐TREM2^−/−^ mice, DIMs rose while DAMs decreased, suggesting that increasing AD symptoms may be associated with an increase in DIM. Keep in mind that these results are only available for mouse species at this time. The process of identifying DAM and its associated subtypes in the human brain is currently underway. The discovery of microglia subtypes highlights the significance of carefully assessing and selectively activating favorable subtypes when developing therapeutics, as research on TREM2 therapy for AD advances.

In the hippocampus and prefrontal cortex of AD mice, Habib et al. identified AD‐associated astrocytes (DAA), which grew in quantity as the illness worsened. It has been shown that DAA works in the early stages of AD by controlling processes linked to the hydrolysis and buildup of Aβ [122]. Additionally, as compared with the homeostatic condition, astrocytes in AD showed a few unique traits. In their scRNA‐seq study of brain samples with a high load of AD pathology, Kun et al. categorized astrocytes. They found that in some subsets of the entorhinal cortex and superior frontal gyrus areas, the expression of genes linked to homeostasis decreased [123]. For example, AD astrocytes downregulated genes linked to the coordination of lipid and oxidative metabolism and shown a lack of metabolic coordination with neurons [117]. Researchers used information from existing AD single‐nucleus sequencing (snRNA‐seq) datasets and spatial transcriptomics resources to provide a more complete picture of AD astrocytes [124]. The data integration made it possible to consistently identify and expand the number of identifiable astrocyte subgroups. They identified new and potentially useful subtypes and positioned them in various levels of the cortex. This discovery highlights the ability to identify hitherto unidentified heterogeneity in neurodegenerative illnesses by merging datasets.

By identifying transcriptional changes in the afflicted areas of the brain, spatial transcriptomics is improving our knowledge of AD. The transcriptional alterations in tissue domains inside a 100 µm diameter of amyloid plaques in an AD animal model are examined using spatial transcriptomics. In the latter stages of the disease, 57 plaque‐induced genes that are linked to inflammation, oxidative stress, the complement system, and lysosomes emerged as a multicellular gene coexpression network. Similar alterations in human brain tissues provide some support for our findings, suggesting that genome‐wide spatial transcriptomics research can provide light on the dysregulated cellular network near the pathogenic hallmarks of AD and other brain disorders [125]. A different research improved the resolution to 55 µm by using the 10× Genomics Visium platform. In conjunction with coimmunofluorescence labeling for pathological markers associated with AD, this technique enabled a comprehensive delineation of gene expression inside the human middle temporal gyrus (MTG), which is known for its early vulnerability in AD. By labeling the white matter (WM) and cortical layers in both AD and normal MTG samples, the researchers were able to identify specific marker genes for the WM and five cortical layers. Additionally, spatial transcriptomics in the olfactory bulb and hippocampus areas of 3×AD, 3×PB, and normal mice were used to investigate the early indicators of AD, such as olfactory impairment and poor short‐term memory. Global dysregulation of genes such as Ubc, Pkm, Cox6c, and Glo1 was discovered in the study, impacting energy generation and cellular metabolism. The importance of these genes in AD pathogenesis and their potential as biomarkers for early AD diagnosis are highlighted by this study [126].

A chronic condition affecting the central nervous system, multiple sclerosis is characterized by localized demyelinating lesions. The presence of reactive astrocytes, stressed oligodendrocytes, and activated microglia in the periphery of MS lesions suggests that glial activation is intimately associated with MS lesions [127]. The various expression patterns and protective roles of microglia in multiple sclerosis have been revealed using scRNA‐seq. Researchers were able to examine the temporal and geographical variability of microglia during both development and disease by utilizing sophisticated single‐cell analysis in conjunction with spatial techniques [128]. One of the genes with the highest levels of overexpression in one of the microglia subgroups was galectin‐1 [129]. It is generally accepted that astrocytes secrete galectin‐1. It can lessen neurotoxicity by encouraging the deactivation of proinflammatory microglia known as classically activated (M1) microglia. These results suggest that this subset of microglia may interact with M1 microglia to modify and control neuroinflammatory responses, so averting demyelination [130]. In light of the increasing attention being paid to myelin dysfunction, a prevalent characteristic of neurodegenerative illnesses including Huntington's disease, AD, and MS, a thorough investigation of microglia and their role in myelin dysfunction is expected in the future.

One prevalent form of neurodegenerative disease that mostly affects the elderly is Parkinson's disease (PD). Its pathogenic characteristics include intracellular α‐synuclein aggregation and the death of dopamine neurons in the brain substantia nigra (SN) [131]. Recent research that combined genome‐wide association analysis and scRNAseq has revealed alterations in oligodendrocyte genetic properties that predate SN degeneration. Additionally, oligodendrocytes expressed genes that were elevated in PD. This finding lends credence to the theory that oligodendrocyte activity or quantity may be a key determinant in the early stages of PD development [132]. However, further research reexamined the significance of neuroinflammation in PD and showed that microglia were heterogeneous. A new subset of microglia was discovered in the midbrain with an immunological alert signature. Its transcriptional characteristics resembled those of microglia activated by inflammation in acute systemic inflammatory situations [133]. Additionally, midbrain samples from single‐cell datasets of idiopathic PD patients revealed a growing proportion of activated microglia. According to trajectory studies, PD‐related inflammation may be caused by a stress response brought on by misfolded microglial proteins [134].

A crucial part of the limbic system in human brains, the hippocampus is involved in both cognitive and spatial navigation. Jia et al. used spatial transcriptomics and scRNA‐seq to identify the patterns of gene expression linked to memory and learning pathways in several geographical areas of the mouse hippocampal region [135]. They discovered that Neurod6, a gene linked to neural development and neuronal survival, is differently expressed. In the setting of PD, they also discovered flag genes for the CA1, CA3, and DG subregions, exposing the transcriptome landscape and heterogeneity of geographically dispersed hippocampus cells. Studies have indicated that PD may develop as a result of changes in the brain's spatial metabolism. Numerous neurotransmitters, such as tyrosine, tryptamine, tyramine, phenylethylamine, dopamine 3‐methoxytyramine, 5‐hydroxytryptamine, γ‐aminobutyric acid, glutamate, and acetylcholine, have been found to exhibit notable variations in abundance in PD, suggesting possible molecular alterations that contribute to the onset and course of the disease [136].

A neurodegenerative disease, amyotrophic lateral sclerosis (ALS) progresses by degenerating motor neurons. Existing evidence has suggested that the superoxide dismutase 1 (SOD1) gene variants are important risk factors [137]. Liu and colleagues identified SOD1 as the primary genetic risk by analyzing the differentially expressed genes (DEGs) of single‐cell populations. They discovered that SOD1 had the most consistent differential expression level across cell types [138]. Necrotic apoptosis of ALS neurons is mediated by receptor‐interacting protein kinase 1 (RIPK1), a kinase that can induce neuroinflammation and axonal degeneration. Researchers discovered a relatively new kind of microglia called RIPK1‐regulated inflammatory microglia (RRIM) in ALS mice models. Inhibition of RIPK1 might effectively limit their prevalence. Classical proinflammatory mechanisms, such as elevated tumor necrosis factor production, were shown to be enhanced in RRIM [139]. Although certain RIPK1 inhibitors have been evaluated in human clinical trials thus far, they have not yet been shown to target particular subtypes of microglia [140]. However, the identification of RRIM may help clarify how (RIPK1) inhibitors are used to treat ALS. This work demonstrates the possibility of using the traits of glial subtypes found by scRNA‐seq to find new regulatory molecules and therapeutic targets [141].

#### 3.2.2 Revealing the Immune Response and Immune Mechanism of Neurodegenerative Diseases

Single‐cell sequencing has become a cutting‐edge method for analyzing pathogenic pathways, examining immune system heterogeneity, and understanding the immunological mechanism in human disorders [142]. Inflammation and immune dysregulation are typical indicators of the majority of dementia. During neurological illnesses, immune cells undergo disease‐specific changes, indicating a critical involvement for these cells in pathogenic pathways. The identification of cellular and molecular targets for diagnosis and therapy will be aided by a better comprehension of gene regulation and particular immune cell types in the immune system [143, 144, 145].

Important new information on the alterations in immune cell gene expression has been made possible by single‐cell transcriptomics analysis. Researchers have developed a more thorough knowledge of the kinds and activities of T cells by utilizing the capabilities of single‐cell TCR sequencing technology [146]. Given the incomplete understanding of the adaptive immune response's function in neurological diseases, these findings have significant implications for the development of immune‐based treatment. As many neurodegenerative disorders advance, the adaptive immune response plays a part. The immunological response mediated by T cells may be linked to the pathogenic advancement in AD. In particular, it has been discovered that T cell infiltration is linked to cognitive deterioration. In AD patients’ cerebrospinal fluid (CSF), clonally increased CD8+ T effector memory CD45RA (TEMRA) cells were detected using scRNA‐seq. These cells were shown to be adversely correlated with cognition, and their increased TCR signals indicate that the adaptive immune response is activated in the blood and CSF of AD patients [147].

Additionally, B lymphocytes contribute to the advancement of neurodegeneration, which can either compartmentalize inside the central nervous system or promote the illness in the periphery. Although MS was once thought to be largely caused by T cells, new research indicates that the disease's progression may be linked to unbalanced interactions between B cells and T cells [148]. T cell proliferation and self‐reactivity were mediated by memory B cells. These T cells, whose quantity may affect the MS lesion region, were verified as brainhoming T cells by T cell receptor deep sequencing. According to a single‐cell transcriptomics, T follicular helper cells encouraged B cell infiltration into the central nervous system, potentially strengthening the autoimmune response. Reducing T cell infiltration in the central nervous system and blocking B cell–T cell interaction may be useful strategies for treating multiple sclerosis [149, 150]. In AD patients, Xiong et al. found a decrease in B lymphocytes, which may be directly linked to the patients’ clinical dementia rating ratings. They discovered that AD genes were the most elevated genes in B cells using scRNA‐seq of peripheral blood mononuclear cells. Cognitive obstacles were exacerbated and the quantity and size of Aβ plaques increased as a result of the early stages of AD's B cell decline. The involvement of B cells in early AD may benefit from more thorough clinical elucidation for early AD diagnosis [151].

### 3.3 The Application of Single‐Cell and Spatial Multiomics in Aging Research

Multiomics is being investigated in the field of aging science in addition to its use in fundamental and clinical disease research. Because aging is a diverse process, various cells experience diseases to varying degrees, which eventually impair the function of organs and tissues. Studying cellular and molecular characteristics linked to aging would shed light on the specifics of the intricate aging process and identify the causes of aging [152]. Cellular studies have been conducted in people and model species to achieve this objective. These studies have used either mixed cell populations or cell populations enriched using recognized markers, both of which can fluctuate and alter with age. Aging phenotypes are heterogeneous between and within tissues and involve multiple molecular levels of regulation, such as epigenetics, transcription, translation, posttranslational regulation, or metabolic regulation.

#### 3.3.1 Application of Single‐Cell Analysis to Aging Neural Systems

The brain uses an integrated network of specialized neurons, glial cells, and endothelial cells to carry out its regular functions. Age does not significantly alter cell identity or composition, according to the single‐cell transcriptome landscape of the aging mouse brain. Cellular respiration, protein synthesis, oxidative stress, inflammatory response, and growth‐factor signaling are the most prevalent aging‐related mechanisms that affect all cell types [153]. Age‐related alterations in microglia in the aging brain are the cause of elevated chronic inflammation. Single‐cell analyses of the brains of Drosophila and mice have shown a slight shift in the composition of the cell population toward microglia in aged brains. These cells are strongly involved in the pathophysiology of age‐related neurodegenerative diseases and undergo changes that exacerbate chronic inflammation as people age [154, 155].

Another area of considerable study and debate is the degree of neurogenesis in older people [156]. Three developmental phases of neural stem cells (NSCs) have been identified by single‐cell analysis of nonneuronal cells isolated from the mouse dentate gyrus. These stages are based on the expression of certain genes. For instance, *Ccnd2*, *Mki67*, *Pcna*, and *Mcm2* are unique for early progenitors, while *Neurod1*, *Sox11*, and *Dcx* indicate late progenitors. Furthermore, the data indicate that the quantity of NSC types rather than their molecular fingerprints is more likely to be impacted by aging [157]. Due to intrinsic processes, a proinflammatory environment and decreased proliferative ability of NSCs and neural progenitor cells have been identified in aged mice brains in another comparable research [158].

One of the hypothesized reasons of aging is the accumulation of somatic DNA mutations. In one work on the subject, genome‐wide somatic SNVs (sSNVs) in DNA from prefrontal cortex and hippocampus neurons were discovered using whole‐genome sequencing at the single‐cell level. Due to hereditary abnormalities in DNA repair, sSNVs build up more quickly in early‐onset neurodegeneration and more slowly with age in the healthy human brain. Therefore, it has been shown that the accumulation of somatic mutations with age represents a molecular signature associated with both age and illness. This signature may now be expanded upon by exploratory studies that examine the probable roles of these mutations [159].

A comprehensive understanding of the aging process across many tissues and cell types has been made possible by large‐scale, multiomics aging atlases. The Tabula Muris Senis project, which provides single‐cell transcriptomic and proteomic data from several mouse organs throughout their lives, is a seminal endeavor in this field [160]. With the use of this extensive dataset, scientists may map transcriptome age predictors and examine the ways in which various cell types within and between organs depart from a “young” transcriptional state. Additionally, these datasets serve as the basis for combining single‐cell data with epigenetic clocks, which are multivariate models that forecast biological age based on DNA methylation patterns (such as the Horvath clock). A more accurate knowledge of the molecular causes of aging can be attained by associating cell‐type‐specific transcriptional alterations with epigenetic age acceleration [161].

Importantly, essential aging indicators may be directly mapped within their tissue context thanks to single‐cell and spatial technology. It is possible to measure and see the expression of senescence markers, such as p16^INK4a (Cdkn2a), and the various elements of the senescence‐associated secretory phenotype (SASP), in many organs [162]. SASP spatial gradients, senescent cell niches, and the proinflammatory effects of these cells on nearby, nonsenescent cells in the tissue microenvironment are all made possible by spatial transcriptomics and proteomics. This method has shown that senescence's load and spatial distribution vary greatly among tissues, which contributes to the functional decline of individual organs [163]. The integration of such multidimensional data from projects such as Tabula Muris Senis is crucial for identifying universal and tissue‐specific mechanisms of aging and ultimately guiding the development of targeted antiaging interventions.

#### 3.3.2 Application of Single‐Cell Analysis to Aging Hematopoietic System

The regenerative and myeloid‐skewed differentiation capabilities of hematopoietic stem cells (HSCs) both drastically decrease with age. Both in the stem cell compartment and in total bone marrow, a scRNA‐seq study has revealed a notable increase in long‐term HSCs (LT‐HSCs) with aging. Meanwhile, multipotent progenitors have been shown to decline with age, whereas the number of short‐term HSCs inside the Lin–Sca1^+^cKit^+^ compartment has been reported to remain constant. LT‐HSCs have been shown to exhibit distinct G1‐S phase depletion by scRNA‐seq analysis. Self‐renewal and differentiation disruption have been shown to have a consistent impact, and the DEGs unique to LT‐HSCs throughout aging have also been discovered [164]. A different scRNA‐seq study of the Lin–Sca1^+^cKit^+^ compartment from mice has revealed that both young and old HSCs exhibit significant DEGs as they mature. A higher percentage of Von Willebrand Factor‐positive LT‐HSCs (Vwf+LT‐HSCs) in aged HSCs has led to the expression of megakaryocyte progenitor (MkP)‐specific genes, according to gene‐set enrichment analysis of these data. Older HSCs with a simultaneous increase of myeloid genes are represented by Vwf^+^ LT‐HSCs, which are platelet‐primed cells. Peripheral blood platelets have been reported to rise in elderly mice in tandem with an increase in MkPs, which is consistent with this observation. Furthermore, the progenitor platelet bias has been linked to the transcriptional regulator FOG‐1. The rise in a unique class of HSCs and the molecular priming of HSCs toward an enhanced myeloid bias with aging have been found by a scRNA‐seq investigation of young and aged HSCs in this dataset. This might lead to age‐associated impaired lymphopoiesis. The power of scRNA‐seq is demonstrated by the fact that alternative approaches would not have been able to provide as comprehensive insight of the change in HSC populations with aging [165].

#### 3.3.3 Application of Single‐Cell Analysis to Aging Immune System

Aside from the change in cell types, the gradual deterioration of physiological and cellular function that characterizes aging is also accompanied by complicated effects on tissue‐specific gene expression and an increase in transcriptional noise, which is the name given to the variation in gene expression within the same cell type [166, 167]. scRNA‐seq analysis of isolated naive CD4^+^ T cells from aged mice has shown that as people age, they lose some of the tight control of the transcriptional program following immune activation. This leads to increased transcriptional variability within a single cell type and a weakened immune response across CD4^+^ T subtypes [168]. B lymphocytes in centenarians had more than 3000 somatic mutations per cell, compared with less than 500 per cell in neonates, according to a single‐cell genomic analysis. The whole mutational landscape of human B lymphocytes suggests that somatic mutations that build up with age may raise an individual's risk of leukemia and functional decrease in B lymphocytes [26]. According to these investigations, spontaneous somatic mutations build up with aging, which may be a factor in the higher risk of age‐related illnesses as well as the functional deterioration seen in cells and organs that can only be identified by single‐cell sequencing. A thorough epigenetic atlas at the single‐cell level has been developed based on the chromatin‐modification patterns in 22 main immune cell subsets using cytometry by time of flight, in addition to transcriptional noise and an increase in somatic mutations. Age‐related increases in interindividual and cell‐to‐cell variability in chromatin marks were found by the chromatin‐modification study, indicating that these alterations may be a factor in the decline of transcriptional control in later life [169].

#### 3.3.4 Application of Spatial Omics in Aging Research

One of the biggest risk factors for the development of chronic kidney disease is aging. The spatial distribution of lipid molecules changed significantly as the kidneys of young and old mice aged, according to MALDI–MSI analysis. The lipid composition of elderly kidneys changed significantly from that of young kidneys, with a rise in ceramides and a decrease in phosphatidylcholines. These results suggest that the functional abnormalities linked to kidney aging are partly caused by lipid changes [170]. One important aging marker accompanying the decline of renal function is renal fibrosis. To comprehend the state of renal illnesses, it is crucial to evaluate their proportion, extent, and spatial distribution. Li et al. used tissue sections that were 600 µm thick and microprobe terahertz time‐domain spectroscopy to get direct spatial resolution of the terahertz spectrum within renal fibrotic tissues. This method provided fresh insights into the diagnosis of fibrosis in various tissue types and allowed for the accurate spatial localization of fibrotic tissues throughout a kidney, especially the distribution of hydroxyproline [171].

One of the main pathological characteristics of diabetic kidney disease and a possible target for treatment is renal fibrosis. In a thorough investigation, glomeruli and fibrotic areas were subjected to spatially resolved transcriptome analysis using scRNA‐seq. The findings showed a strong correlation between venous endothelial cells (VECs) and renal fibrosis, with VECs accumulating in renal fibrotic regions inside nephropathy‐affected tissue. Additionally, there was a clear geographical association between the prevalence of immune cell populations in these areas and renal fibrosis [172]. These discoveries broaden our knowledge of the cell populations, interactions between cells, and changes in signaling pathways in regions affected by renal fibrosis.

Loss of muscle mass and the disintegration of muscle fibers are hallmarks of skeletal muscle aging, a common phenomenon seen in all animals [173]. Age‐related anatomical alterations frequently result in impaired muscle function, which causes problems such muscle weakness, slowed mobility, and diminished endurance. In two different Duchenne muscular dystrophy mice models with differing disease severity, Dutch researchers found gene expression patterns linked to skeletal muscle pathology and established a clear association between gene expression and muscle histology [174]. Increased expression of particular genes was found by spatial analysis in areas linked to calcification (*Bgn*, *Ctsk*, *Spp1*), fibrosis (*Vim*, *Fn1*, *Thbs4*), and muscle regeneration (*Myl4*, *Sparc*, *Hspg2*). The difficulties of associating transcriptional changes in the spatial dimension with histological changes were effectively overcome by this method. Muscle degeneration brought on by age is frequently linked to shoulder pain and restricted range of motion. Researchers used spatial metabolomics on human rotator cuff muscles to determine the biochemical signature of aging muscle tissue. They discovered that different kinds of muscle fibers have different lipid enrichments. Degenerated areas had 37 distinctive ions, with heme being the most prevalent metabolite. Healthy parts displayed 49 unique ions. For chronic shoulder muscle degeneration, these characteristic metabolites may act as markers of muscle deterioration and direct individualized therapy [175]. Leg muscles from young and old men with and without metabolic syndrome were metabolically imaged in another investigation. After identifying the fiber types present in ROI through biopsies, MALDI–MS imaging found that the fat content of the various fiber types was very comparable. On the other hand, some lipid droplets, such C14 sphingosine or lauroyl ethanolamine, can distinguish between muscles that are aging or have metabolic syndrome [176].

Ovarian aging, which lowers follicle count and oocyte quality and eventually impacts reproductive capacity, is a defining feature of the aging process within the female reproductive system. Oocytes, granulosa cells, and stromal cells are among the several cell types that make up the ovary, a complex and diverse organ. Each of these cell types has a unique role that adds to the organ's overall heterogeneity [177]. Eight main ovarian cell types and their various subpopulations were identified in one study by spatial transcriptomics mapping the ovaries of young and aged mice [178]. Notably, several granulosa cell subpopulations were distinguished in the antral follicles, separated into inner and outer subpopulations according to their spatial shape. These subgroups express distinct transcripts, such as *Sohlh1*, *Zar1*, and *Nobox*, which are essential for early follicle activation and development, and their gene expression profiles are markedly different. Additionally, granulosa cells with different spatial‐gene‐expression patterns were also found in a different investigation that compared the ovaries of young and old rats [179]. Three subtypes of granulosa cells, each representing a successive developmental stage, were revealed through the dynamics of gene expression. Moreover, it has been established that granulosa cell oxidative pathway disruptions are important contributors to ovarian aging. These results highlight the intricacy of age‐related ovarian cell population alterations as well as the significance of spatial morphology and gene expression patterns in comprehending ovarian aging.

Recent research has demonstrated the important influence that balanced follicle metabolism has on several facets of nuclear and cytoplasmic maturation, as well as the embryos’ subsequent developmental competence [180]. Researchers have used MALDI imaging to examine oocytes at different developmental stages and aging in order to clarify the metabolic landscape of the aging reproductive system. This has made it easier to identify changes in lipid composition and localization linked to different oocyte functions [181]. Particularly, the results specifically showed that oocytes were rich in phosphatidylcholine, phosphatidylinositol, and sulfate.

In conclusion, it has become clearer that spatial omics technologies have the potential to offer important insights into the complex molecular changes that define the aging brain as a result of extensive research in the field of brain aging research using these technologies. Furthermore, their use can be expanded to include aging, neurodegenerative disorders, and HNSCC, providing vital information for better comprehending the mechanisms behind these conditions and creating possible treatment approaches.

## 4 Integrating Single‐Cell and Spatial Multiomics Data—Using Deep Learning Approaches

The remarkable resolution in the investigation of cell heterogeneity at various omics levels is made possible by the fast advancement of single‐cell and spatial multiomics. One of the core topics in biology is figuring out how individual cells interact with one another and react to changes in their surroundings [182]. Differences in the composition of various cell types may be hidden when studying large populations of cells due to cell heterogeneity. On the other hand, single‐cell and spatial multiomics analysis can make it possible to investigate biological systems in a more methodical manner, exposing fundamental mechanisms of cellular functioning and biological processes such as cell differentiation and disease development. Since single‐cell isolation and sequencing techniques have advanced over the past decade, it is now possible to assess DNA, mRNA, and protein abundances at the single‐cell level. For example, individual cell types and functional states can be determined by single‐cell transcriptomics and proteomics studies, whereas genetic variability within individual cells can be shown by single‐cell genomic analysis [183]. However, it is inherently difficult to analyze single‐cell data of enormous dimensions and size due to the quantity of noise, sparsity, batch effects, and unpredictability in the data [184]. ​Therefore, developing new computational models that can handle the dimensionality, size, and other features of single‐cell and spatial multiomics data is essential for deep learning.

### 4.1 Deep Learning Approaches

In the deep learning approach, recurrent neural network (RNN) is used to interpret sequential data, including time series and spoken language. While processing sequential inputs one at a time, RNNs implicitly record the input sequence's prior elements [185]. RNNs acquire crucial data by recurrently propagating the gradients of the inputs to every hidden state. It can be difficult to train RNNs since backpropagation might cause the gradient to disappear or explode. The sustainable and comprehensible RNN approach involves the hidden units producing different outputs at different time iterations, which are the result of numerous neurons in a deep multilayer network [186].

A unique method for identifying possible targets for transcription factors is to use RNN for simulation in time series gene expression data. RNN may be applied as a biological process simulator to investigate gene activity inside the single‐cell analysis's effective time zone [187]. Another study employed the dual‐stage attention‐based RNN (DA‐RNN) for time series prediction using a deep neural network model. It predicts the behavior of a target variable in a stochastic ensemble of time traces to estimate a set of proteins’ concentrations. The DA‐RNN model was trained using onetime series including 1001 time points for every gene regulatory network. The time window for training the network was then determined by assessing the DA‐RNN accuracy and the autocorrelation time of the gene expression time traces [188]. In general, determining transcription factor targets and creating a gene regulatory network can be facilitated by an RNN model trained on time series gene expression data. The result demonstrates that the enhanced RNN method outperforms most other methods in terms of accuracy when it comes to finding transcription factor targets.

Convolution is a mathematical process that is used in place of tensor multiplication, which is what FFNNs perform, at different layers in the convolutional neural network (CNN) approach [189]. CNN is very important for processing data with grid‐like topology. Two major issues arise from the fact that CNNs perform better than conventional image processing techniques in terms of computation speed and performance. CNN must first be able to quantify the characteristics of cells. Second, when a vector list of cell measurement sizes depends on the total number of cells in the picture, the CNN output has finite dimensions [190]. CNNs were demonstrated to be able to hand deep learning a variety of cell measurements on a range of cell pictures with different cell concentrations and image quality. A single CNN model can be trained to replace the entire image processing pipeline and perform cell measurement quantification [191]. Self‐learned hierarchical features are unbiased and can provide new insights into the key structure of class discrimination. In addition to the high accuracy of the CNN method, it can offer other advantages [192].

Generative adversarial networks (GANs) in bioinformatics offer a number of benefits over traditional generative models, such as the ability to learn any distribution without requiring any previous distribution assumptions and the lack of latent space size limitations [193]. The discriminator network is taught to discern between real and synthetic samples in the GNN model, where the generator network generates a series of repeated samples that mimic the distribution of real data. Learning the nonlinear gene‐gene correlations from complicated, multicell type data are the basic idea behind scIGANs, which is to train a generative model with the actual expression of specified cell types [194]. The primary benefit of the GNN model is that, although it lacks the capacity to adequately represent uncommon cell types, it avoids overfitting for cell types in huge populations [195]. Another study demonstrates how biological data from multiple laboratories can be used to evaluate a novel generative deep learning technique employing GNN on scRNA‐seq data from epidermal, brain, and hematopoietic tissues. It was demonstrated that several scRNA‐seq datasets could be combined and that the generative model could precisely reproduce scRNA‐seq data that take different cell types into consideration [196]. Overall, GNNs are highly capable of learning and reproducing any data distribution. With several versions, GANs have gained popularity as a study topic and show promise for data imputation.

Although general‐purpose deep learning architectures serve as a basis, the field has quickly developed to incorporate specialized models created to handle the unique difficulties of single‐cell and spatial data, including high dimensionality, sparsity, technical noise, and the integration of spatial coordinates. ScVI (single‐cell variational inference) is a fundamental probabilistic framework based on variational autoencoders for single‐cell transcriptomics. It facilitates the study of big datasets with millions of cells by performing batch correction, denoising, and nonlinear dimensionality reduction in a unified manner [197]. Building on scVI, single‐cell annotated variational inference is a semi‐supervised model that uses cell‐type labels to accomplish greater integration and label transfer. It is especially useful for annotating novel cell states and harmonizing datasets with complex batch effects [198].

Graph neural networks (GNNs) are the preferred model for spatially resolved data because they can represent spatial neighborhoods as graphs. GraphST is a typical spatial GNN that successfully combines gene expression and spatial location to carry out denoising, spatially aware clustering, and the identification of spatially variable genes. It has proven to be quite effective for defining the architecture of complicated tissues, such as the mouse brain and samples of breast cancer [199]. Another effective method, SpaGCN, uses spatial transcriptomics data and the histological picture to achieve precise cell‐type deconvolution and find geographic areas with coherent expression and histology. It has been effectively employed to map the course of AD in human postmortem brain slices and identify new tissue niches in glioblastoma, demonstrating correlations between certain glial subpopulations and clinical characteristics [200]. The development and application of these specialized models are crucial for extracting biologically meaningful insights from the ever‐growing volume of single‐cell and spatial multiomics data generated in both health and disease.

### 4.2 Deep Learning‐Based Integrative Analysis of Single‐Cell and Spatial Multiomics Data

Every advancement in omics techniques requires the development of sophisticated omics analysis tools to facilitate the analysis and comprehension of the collected multidimensional data. It is now possible to process large amounts of high‐throughput research data from many observational sources in an effective manner. When analyzing and interpreting single omics data, the disregard of crosstalk between various molecular entities results in the loss of related biological information. Multiple omics data collection from a single sample offer a rare chance to gain a more thorough understanding of the information flow between biological levels. The term “data integration” is widely used in a variety of different omics approaches, including horizontal data integration, which combines identical omics data from several studies or time periods [201, 202]. A procedure known as vertical data integration involves combining several omics data types that were taken from the same sample. Often called “multiomics integration,” vertical data integration are a highly complex subject and one of the main focuses of current study.

Early integration and late integration are the two methods used in multiomics integration. Early integration is the process of concatenating all omics data before to analysis, which may highlight relationships between variables from different level [203, 204]. Following separate querying of a single dataset, late integration entails conducting a comparison analysis, such as detecting disruptions in shared biological pathways. Late integration approaches are likely more commonly used than early ones due to the difficulties in combining raw data from several modalities [205]. Integrated methods for simulating biological information flow between different data types are feasible, and the development of integrated methods is a rapidly deep learning growing area of research.

In general, there are two types of techniques to integrating omics: machine learning and statistics. Matrix factorization (MF) and univariate correlation are examples of statistical techniques. Univariate correlation is the process of examining the connection between a phenotypic outcome and each omics parameter (such as a gene, protein, or genetic variant) [206, 207]. Several testing changes are required to account for false positives since these procedures often produce large false positive rates. An unsupervised learning technique called MF may be used to reduce the number of dimensions in omics datasets while preserving the most information possible for additional analysis. Two representative matrices may be produced with MF, one of which summarizes the dataset's attributes and the other of which shows the connections between the samples [208].

Network‐based techniques include network analysis using software such as weighted gene correlation network analysis and network visualization using web‐based tools like PaintOmics [209, 210]. In a network, nodes represent biological elements such as proteins or metabolites, while edges display the current relationships between two nodes. Numerous methods may be used to examine networks produced from omics data. Each network may be utilized to create a multilayer network model, or all of the omics‐specific networks can be fused into a single network using a fusion technique. Learning the characteristics of each network and using them as the input for a classification or prediction model is an option [203, 211, 212]. By making it possible to identify and examine connections within and across omics layers, the creation of multiomics networks offers a thorough knowledge of a biological system [213].

**FIGURE 3:**
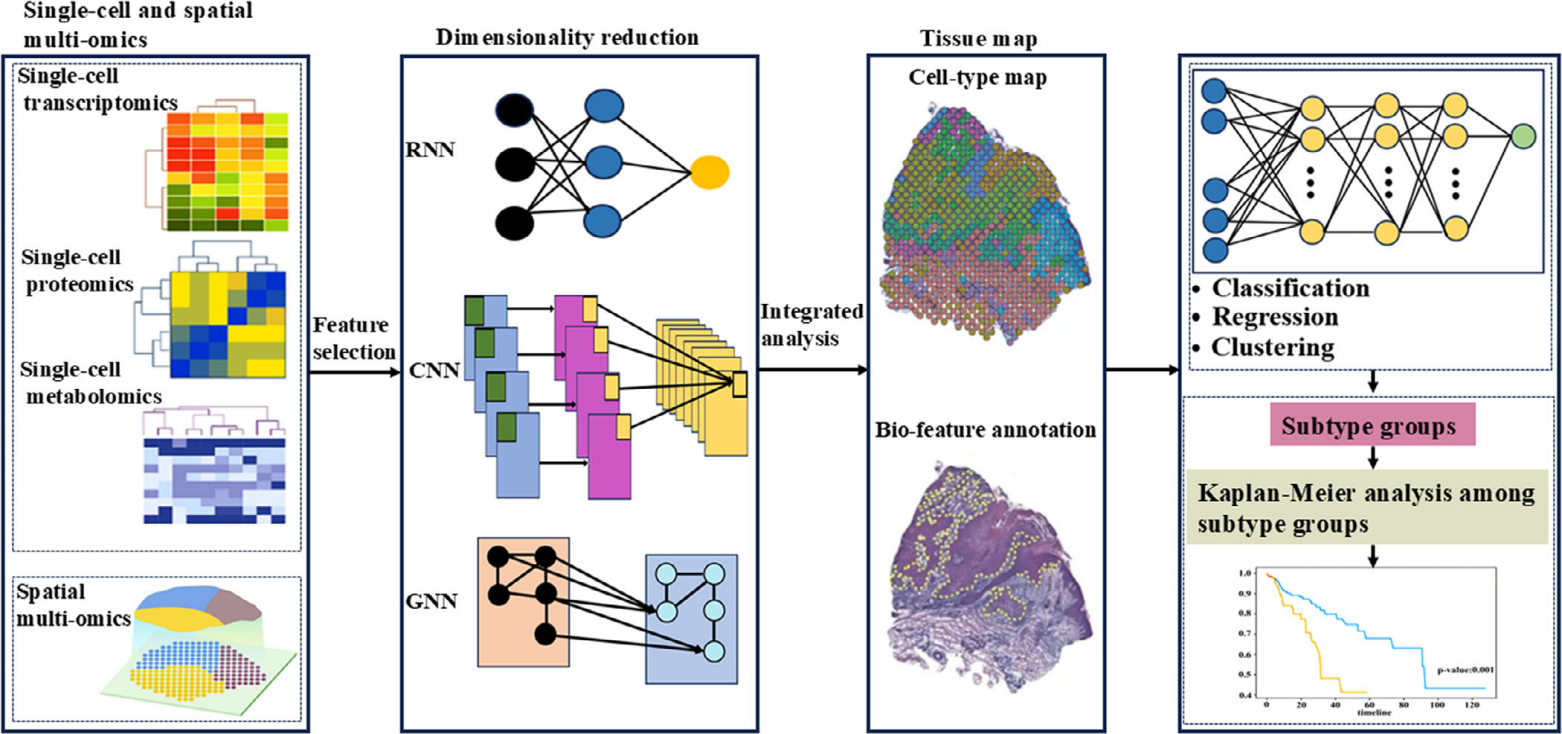
Workflow for single‐cell and spatial multiomics ensemble approaches (by FigDraw). It explains how integrating multiomics might aid in the understanding and treatment of diseases, and shows how deep learning techniques can be integrated at the single‐cell and spatial multiomics levels.

Deep learning algorithms have become one of the most promising methods for assessing multiomics data due to their ability to capture hierarchical and nonlinear properties as well as their prediction accuracy. These methods, which are based on the neural network concept, may be used to identify nonlinear and hierarchical features in data [214]. Based on the deep learning approach, multiomics approaches can aid in drug repurposing, disease subtypes, and identification of new biomarkers. The multiomics approach has been recommended as a promising tool to gain a deeper understanding of the HNSCC process and its underlying causes (Figure 3).

When utilizing multiomics high‐dimensional, low sample size data, it is much more difficult to train deep learning models with numerous parameters without overfitting and bias [215, 216]. By avoiding overfitting during the backpropagation phase, a leave‐one‐out technique can assist in resolving the high‐dimensional, low sample size problem in deep learning model training [217]. When overfitting happens, the training can be stopped early using the leave‐one‐out technique, which is used to confirm the danger of overfitting in the data check. To mitigate the overfitting risk with validation and reduce the backpropagation gradients with high variation on high‐dimensional, low sample size data, it is often advised to use a moderate learning rate and severe dropout regularization with higher epoch sizes. Another strategy has been to try to manageably lower the dimensionality of the input space by placing a random item layer in front of the network [218, 219]. There are two methods for interpreting models in deep learning: (1) interpretation founded on the intrinsic model and (2) interpretation based on posthoc analysis [220]. Intrinsic model interpretation is the process of reading the model directly from its development and the optimal parameters of deep learning models to understand the relationships between the biological components from the data. For instance, neural networks with sparse connections have shown the ability to read internal models. Hierarchical links between subsets of biological components in multilayer biological processes may be found by integrating biological route databases into their models [221].

Artificial intelligence is increasing in importance in the context of “big data” mining, particularly in precision medicine. Particularly in the single‐cell community, deep learning approaches have drawn a lot of interest because of their adaptability to a variety of applications and their capacity to hand deep learning diverse, sparse, noisy, and high‐dimensional single‐cell omics data [222]. For example, deep learning approaches have been shown to be highly effective in tasks such as cellular trajectory inference, batch effect removal, data imputation, and dimensionality reduction [223, 224]. Deep learning approaches have a reputation for being difficult to interpret, especially when it comes to comprehending the underlying molecular mechanisms that underlie cellular functions and phenotypes [225]. Therefore, there has been a growing focus on making models more interpretable, particularly for applications like identifying molecular control and reassembling biological networks [226, 227, 228].

## 5 Single‐Cell and Spatial Multiomics Toward Precision Medicine

Precision medicine, also termed customized therapeutics, aims to optimize clinical management through patient‐specific diagnostic and treatment strategies underpinned by multiomics profiling. The conceptual evolution of this approach gained significant momentum following the 2015 Precision Medicine Initiative introduced during the Obama administration, particularly addressing the critical need in oncology where approximately 30–50% of patients exhibit suboptimal therapeutic responses to conventional pharmacotherapy [229]. This paradigm shift from empirical treatment to biomarker‐driven care enables clinicians to implement targeted interventions based on individual molecular signatures, fundamentally redefining therapeutic decision‐making processes. While the foundational principle emphasized pharmacogenomic optimization to enhance drug safety and efficacy, contemporary implementations increasingly integrate proteomic, metabolomic, and environmental data to construct comprehensive therapeutic algorithms.

Personalized medicine using multiomics techniques has emerged as the new standard for medical treatment. While personalized therapies have gained popularity due to genome sequencing advancements, clinical decision‐making has been influenced by environmental variables and other molecular profiling techniques that evaluate the transcriptome, metabolome, proteome, and epigenome. Combining multiomics profiles into a single measure for characterizing health and disease is now feasible because to advancements in computational methods and device technology [230, 231]. These multiparameter patient profiles often produce significant amounts of data that allow for extremely accurate patient categorization, biomarker identification, and functional drug kinetics. Patient subtyping is the cornerstone of personalized treatment [203]. The majority of multiomics research in clinical settings is based on conventional ensemble level data. Conversely, subcellular responses determine customized treatment options, and cellular interactions inside tumors govern cancers. Cellular phenotypes linked to disease and their abundances in different forms of cancer have been revealed using single‐cell mass cytometry and single‐cell sequencing techniques [22]. These methods use cellular suspensions created from patient‐derived blood and sorted cells to examine drug resistance in treatments. Single‐cell variations revealed the principles of tumor compositions and specific immune subgroups that differ in different cancers. For example, lung adenocarcinoma, a new cellular phenotype for immunotherapies, demonstrated cytolytic activity in NK cells [232]. The spatial range of cellular interactions is still a missing component of these single‐cell approaches. This viewpoint presents spatial‐omics technologies that are perfect for creating customized therapies [233]. In patient specimens, single‐cell features and their local interactions are captured by spatially resolved proteomics, genomes, and metabolomics techniques. The final genetic profiles of resistant and responsive patient groups will be provided by spatial multiomics approaches in conjunction with patient features.

**FIGURE 4:**
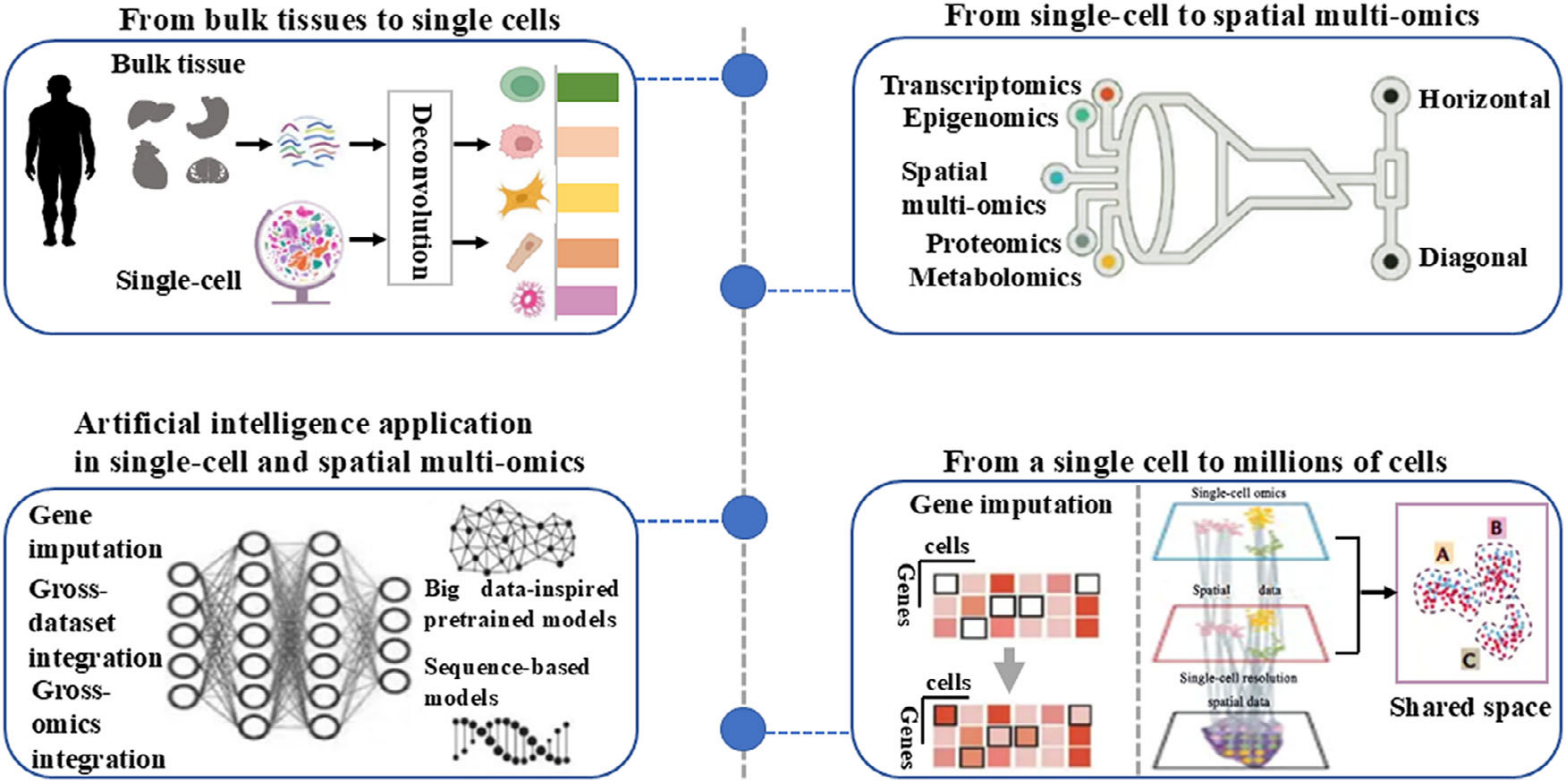
Workflow for single‐cell and spatial multiomics ensemble approaches to deep learning (by FigDraw). The research introduces deep learning‐based data integration studies using different omics data, where most related research belongs: (1) feature selection/reduction, (2) clinical outcome prediction, (3) survival analysis and (4) clustering for subtype discovery. *Abbreviations*: CNV: copy number variation; SNP: single nucleotide polymorphism.

One area of computer science is artificial intelligence. It creates and uses sophisticated mathematical and computational techniques to “learn” intricate clinical data structures and “predict” treatment signs from datasets based on quantifiable features, opening up several possibilities for precision medicine [234, 235]. An essential component of personalized medicine is to model the complex diversity of individuals through patient stratification. The advent of machine learning and deep learning frameworks has made it possible to subtype patients based on pathology maps, molecular imaging data, and molecular data (genomics, proteomics, and metabolomics) [236]. Although all datasets are from the same patient, it is still difficult to combine such multiomics data with various attributes. These data formats are incompatible, which negatively impacts patient categorization performance. Spatial multiomics techniques provide great sensitivity and direct visualization of transcript, protein, and metabolite profiles to solve these problems and perhaps enhance patient categorization. Spatial profiles are consistent between individuals or between patient cohorts because they are well aligned at the cellular level. The magnitude of the datasets is a major obstacle for medical machine learning. While the findings of genome‐level sequencing are useful, huge molecular maps at the single‐cell and subcellular levels are produced using spatial single‐cell data. The performance of automated patient group stratification will therefore be improved by the cellular large data produced by the spatially resolved analysis. Drug response to a particular therapy may then be coupled with intelligent patient classifications that are generated from spatial multiomics platforms. As the first phase in the precision oncology framework, this detailed data on patient groups and their linkage to molecular subtypes of patients will be used as “training” for machine‐ and deep‐learning algorithms. Particularly in radiology and pathology, artificial intelligence algorithms have been used to predict patient treatment outcomes based on imaging data and genetic mapping. Preprocessed PET scans, which comprise quantitative data assessed by CNNs in esophageal cancer, are used to predict the response to neoadjuvant treatment [237, 238]. To forecast patient survival patterns, spatial distance measurements in MRI pictures of patients with glioblastoma multiforme tumors were employed. The likelihood of recurrence was also predicted using a classifier system that evaluated tumor‐infiltrating lymphocytes in pathology images [239]. Clinical outcome predictions were made possible by combining molecular genome‐wide data from thousands of patients with pathological spatial characteristics [240]. Furthermore, cellular developmental abnormalities were included into a machine‐learning model (regularized elastic‐net technique) to predict the recurrence of leukemias, and multiplexed single‐cell proteomic profiles were evaluated in pediatric diseases [241]. In the future, molecular imaging bioimaging characteristics (cellular locations, interaction frequencies, tissue structural changes, and subcellular distributions) and single‐cell analysis combined with spatial multiomics approaches may significantly influence HNSCC precision medicine prediction outcome studies (Figure 4).

## 6 Conclusions and Prospects

Technological advances have ushered in the era of single‐cell omics, allowing the collection and integration of data from individual cells. In biological studies of HNSCC, it is critical to identify biomarkers of HNSCC and targets for HNSCC therapy. Single‐cell and spatial multiomics data can be used to develop innovative ways to diagnose and treat HNSCC, as well as to better understand the disease process. Above all, the established methods of single‐cell and spatial multiomics have the potential to fundamentally change future medical practice.

The biological significance of various cell subtypes in certain disorders can be better understood by researchers using single‐cell omics approaches. Single‐cell omics techniques make it possible to comprehend the complex interactions between different cells and their subtypes, as well as the role these cells play in a person's immune response. The pathogenic alterations and intercellular connections of some diseases can be shown with the use of spatial omics technology, which would enhance disease diagnosis and therapy. Clinical applications of these technologies have been used to identify and treat diseases like breast and lung cancer. To increase diagnostic accuracy and treatment outcomes, single‐cell and spatially multiomics techniques will be used more for cancer tailored diagnosis and treatment.

However, there are several problems and challenges associated with the use of single‐cell and spatial multiomics techniques. First, one of the main problems with single‐cell omics techniques is the complexity of data processing and interpretation. Second, despite the fact that spatial multiomics approaches are capable of properly describing the spatial position and distribution of many cell types in tissues, some cell types and molecular distributions remain difficult to detect and identify with present technologies, which may limit their application in clinical diagnostics. Consequently, more technology advancements and optimizations are required. High implementation costs, which include investing in related analytics and algorithms as well as buying, maintaining, and running equipment, are another issue with the use of single‐cell and spatial multiomics technologies. When using this method, it is also important to take into account the time and expense involved in doing single‐cell analysis on many patients. Data security and personal privacy are additional concerns that require consideration. Since genomes and biomarkers are examples of private personal data that are involved in single‐cell and spatial multiomics methods, more robust and trustworthy security measures are required to safeguard patient and participant rights and privacy. Because single‐cell and spatial multiomics methods are critical to gaining a thorough understanding of the cellular kinds, metabolic, genetic, and molecular networks of HNSCC, they have garnered a great deal of interest in personalized and precision medicine [242, 243]. Therefore, we recommend conducting a comprehensive study using single‐cell and spatial multiomics data to objectively describe the course of HNSCC.

The development of computer science, such as artificial intelligence, will accelerate the study of HNSCC biomarkers and mechanisms. Despite the significant gap between biomarker discovery and clinical application, single‐cell and spatial multiomics data analysis is a promising approach for discovering new biomarker candidates, identifying therapeutic targets, and facilitating a comprehensive understanding of complex HNSCC pathophysiological processes. By capturing intricate nonlinear effects, hierarchical characteristics, and their interactions in multiomics data, deep learning is a successful method for deciphering multilayered complicated biological systems. In particular, big data‐driven and objective deep learning techniques may now collect comprehensive molecular data to deconstruct and find patterns in the data and offer more understanding of the biology of diseases as well as the health conditions of specific individuals. But when comparing the current literature to new high‐throughput technologies, it is clear that there has been little integration of multiomics data. Even fewer reports describe the multiomics integration approach with clinical environmental data, standards, or ground truth to assess the performance metrics of multiomics integration methods to clarify their role in health and disease.

Deep learning methods are expected to improve patient prediction, prevention, diagnosis, and tailored therapy by using multiomics data. As the study progresses, taking into consideration racial differences in lifestyle and environmental exposure, as well as a clinical and multiomics‐based data‐driven deep learning framework with integration, will be essential for discovering patterns and developing risk detection and prediction methods for particular clinical outcomes. Therefore, it is crucial to have fast algorithms that are robust against heterogeneity and missing data and provide a reasonable trade‐off between speed and model interpretability. These technologies will support the creation of tailored, precise treatments for complex diseases.

Integrating multiple omics data can provide synergies and molecular insights that exceed the sum of individual omics. How can we choose interpretable features? is one of the many concerns that need to be addressed as the study progresses since interpretability is more crucial than accuracy in deep learning modeling. What characteristics, and how, go into making a certain prediction? and how can a black‐box model be explained in terms of biology or medicine? There will be widespread use of multiomics data integration techniques for precision medicine and a thorough disease risk assessment.

## Author Contributions

**Wentao Li**: wrote the paper draft. **Chao Cheng**: edited the paper draft. **Xin Zhu**: supervision and funding acquisition. **Chenping Zhang**: conceived the project and funding acquisition. All authors have read and approved the final manuscript.

## Ethics Statement

The authors have nothing to report.

## Conflicts of Interest

The authors declare no conflicts of interest.

## Data Availability

The authors have nothing to report.
